# Effects of olmesartan on arterial stiffness in rats with chronic renal failure

**DOI:** 10.1186/1475-2840-11-66

**Published:** 2012-06-13

**Authors:** Yao-Chen Chuang, Ming-Shiou Wu, Yi-Kai Su, Kwang-Ming Fang

**Affiliations:** 1 Institute of Physiology, College of Medicine, National Taiwan University, 10 F, No. 1, Sec., 1, Jen-Ai Road, Zhongzheng Dist., Taipei City, 100, Taiwan; 2 Department of Internal Medicine, National Taiwan University Hospital, No.7, Chung Shan S. Rd., Zhongzheng Dist., Taipei City, 100, Taiwan; 3 Department of Dental Technology and Materials Science, Central Taiwan, University of Science and Technology, No.666, Buzih Road, Beitun District, Taichung City, 40601, Taiwan

**Keywords:** Advanced glycation end products, Aortic impedance analysis, Chronic renal failure, Diabetes

## Abstract

**Background:**

It has been suggested that the antioxidant properties of olmesartan (OLM), an angiotensin II type 1 receptor (AT_1_R) blocker, contribute to renal protection rather than blood pressure lowering effects despite the fact that causal relationships between hypertension and renal artery disease exist. This study aimed to examine the hypothesis whether the antioxidative activities of OLM were correlated to arterial stiffness, reactive oxygen species and advanced glycation end products (AGEs) formation in rats with chronic renal failure (CRF).

**Methods:**

CRF rats were induced by 5/6 nephrectomy and randomly assigned to an OLM (10 mg/day) group or a control group. Hemodynamic states, oxidative stress, renal function and AGEs were measured after 8 weeks of OLM treatment.

**Results:**

All the hemodynamic derangements associated with renal and cardiovascular dysfunctions were abrogated in CRF rats receiving OLM. Decreased cardiac output was normalized compared to control (*p* <0.05). Mean aortic pressure, total peripheral resistance and left ventricular weight/body weight ratio were reduced by 21.6% (*p* <0.05), 28.2% (*p* <0.05) and 27.2% ((*p* <0.05). OLM also showed beneficial effects on the oscillatory components of the ventricular after-load, including 39% reduction in aortic characteristic impedance (*p* < 0.05), 75.3% increase in aortic compliance (p <0.05) and 50.3% increase in wave transit time (*p* < 0.05). These results implied that OLM attenuated the increased systolic load of the left ventricle and prevented cardiac hypertrophy in CRF rats. Improved renal function was also reflected by increases in the clearances of BUN (28.7%) and serum creatinine (SCr, 38.8%). In addition to these functional improvements, OLM specifically reduced the levels of malondialdehyde (MDA) equivalents in aorta and serum by 14.3% and 25.1%, as well as the amount of AGEs in the aortic wall by 32% (*p* < 0.05) of CRF rats.

**Conclusion:**

OLM treatment could ameliorate arterial stiffness in CRF rats with concomitant inhibition of MDA and AGEs levels through the reduction of oxidative stress in aortic wall.

## Background

Increased arterial stiffness is associated with the development and progression of chronic kidney disease (CKD) [1,2]. The accumulation of advanced glycation end products (AGEs) due to reduced capability of detoxification and excretion in CKD patients has been confirmed to worsen vascularpathy [3,4]. AGEs stiffen collagen backbones [5], promote collagen deposition in heart and aorta [6], increase the expression of growth factors and cytokines [7] and induce inflammation [8]. These products can consequently lead to glomerular and tubulointerstitial injury. Satisfactory glycermic control, which can reduce AGE accumulation, has been shown effective on lowering arterial stiffness in diabetic patents [9]. Angiotensin receptor blockers (ARBs) exhibit pleiotropic effects that prevent vascular stiffness [10-12]. Previous studies have demonstrated that ARBs have renoprotective effects to reduce diabetic nephropathy and complications, such as hyperfiltration, increased intraglomercular pressure and urinary protein excretion, in patients with or without diabetes [13,14]. ARBs reduced AGE formation by blocking excess oxidative stress has been verified in vitro [7]; however, the protective effect of ARBs on AGE formation remained controversial in clinical study [15].

The effects of Olmesartan (OLM), a newest ARB, on oxidative stress have been confirmed in a clinical study of patients with diabetes [16]. Although it was suggested that the antioxidant properties of OLM contribute to renal protection rather than BP lowering effects [17], the causal relationships between hypertension and renal disease exist [18,19]. A recent report indicated that OLM decreased the time to the onset of microalbuminuria in patients with type 2 diabetes [20]. Therefore, this study aimed to examine the hypothesis whether the antioxidative activities of OLM were correlated to arterial stiffness, reactive oxygen species (ROS) and AGEs formation by using 5/6 nephrectomy-induced CRF rats. Besides, we also evaluated the effect of OLM on the renal function by the measurements of serum creatinine (SCr) and blood urea nitrogen (BUN).

## Methods

### Experimental Animals

Male Wistar rats (8 weeks old) were randomly divided into three groups; (1) normal controls (NC), (2) CRF, and (3) CRF treated with OLM (CRF + OLM). All rats were allowed free access to water and chow (Rodent Diet 5001, PMI Lab, St. Louis, MO, USA) and housed in an animal room with a 12-h light/dark cycle. All animal experiments were conducted in accordance with the Guide for the Care and Use of Laboratory Animals and the protocols were approved by the Animal Care and Use Committee of the National Taiwan University. The rats were allocated randomly to 3 groups of 15 each: (1) normal control (NC), (2) chronic renal failure (CRF), and (3) OLM-treated CRF rats (OLM + CRF).

### 5/6 Subtotal Nephrectomy

Rat 5/6 subtotal nephrectomy (SNx) is a classic animal model of CRF with enhanced activity of the renin-angiotensin system. In this study, CRF was induced by 5/6 subtotal nephrectomy as previously described [21]. Under anesthesia with sodium pentobarbital (50 mg/kg; i.p.), two branches of the left renal artery were ligated to create an infarction after right nephrectomy. Rats in the normal control group underwent sham surgeries. After recovering for one week, CRF rats received daily oral gavage with OLM (10 mg/kg/day) or placebo for the next 8 weeks.

### Aortic input impedance spectra

General surgical procedures and measurements of hemodynamic variables in anesthetized rats were described before [22]. Briefly, after anesthesia and intubation, the rats were placed on a heating pad and ventilated with a rodent respirator (Model 131; New England Medical Instruments, Medway, MA). The chest wall was opened through the right second intercostal space. An electromagnetic flow probe (Model 100 series, internal circumference = 8 mm; Carolina Medical Electronics, King, NC) was positioned around the ascending aorta to measure the pulsatile aortic flow. A high fidelity pressure catheter (Model SPC 320, size = 2 F; Millar Instruments, Houston, TX) was used to measure the pulsatile aortic pressure via the isolated carotid artery on the right side. An electrocardiogram from lead II was recorded with a Gould Electrocardiograph/Biotech amplifier (Gould Electronics, Cleveland, OH).

The selected pressure and flow signals of 5–10 beats were averaged in the time domain using the peak R wave of the electrocardiogram as the fiducial point. Because of the spatial distance between the flow probe and proximal aortic pressure transducer, timing between the pressure and flow signals was corrected by a time-domain approach, in which the foot of the pressure waveform was realigned with that of the flow [23]. The resulting pressure and flow signals were subjected to further vascular impedance analysis.

The aortic input impedance was obtained from the ratio of the ascending aortic pressure harmonics to the corresponding flow harmonics, using a standard Fourier series expansion technique [22,24,25]. Total peripheral resistance of the systemic circulation was calculated as the mean aortic pressure divided by the mean aortic flow rate. The aortic characteristic impedance was computed by averaging the high-frequency moduli of the aortic input-impedance data points (4^th^–10^th^ harmonics) [26,27].

After taking the aortic characteristic impedance into consideration, we calculated the systemic arterial compliance (C) at the mean aortic pressure (P_m_) by expanding the two-element Windkessel model into a three-element model [28], which accounted for the nonlinear exponential pressure-volume relationship:

(1) $$C(P_{m})=\frac{SVb}{K+Z_{C}SV/Ad}\times \frac{e^{b\times P_{m}}}{e^{b\times P_{i}}-e^{b\times P_{d}}}\text{,}$$

where *SV* is the stroke volume, *K* is the ratio of total area under the aortic pressure curve to the diastolic area (*Ad*), *Z*_*c*_ is the aortic characteristic impedance, *b* is the coefficient in the pressure-volume relationship (-0.0131 ± 0.009 in the aortic arch), *P*_*i*_ is the pressure at the time of incisura, and *P*_*d*_ is the end-diastolic pressure.

The wave transit time can be computed by the impulse response of the filtered aortic input impedance. This was achieved using an inverse transformation of aortic input impedance after multiplying the first 12 harmonics by a Dolph-Chebychev weighting function of the 24^th^ order [29]. Then, the time-domain reflection factor was derived as the amplitude ratio of the backward-to-forward peak pressure wave using the method proposed by Westerhof *et al*. [30]. Thus, both the wave transit time and the wave reflection factor characterized wave reflection phenomena in the vasculature.

### Biochemical analysis

After 8 weeks, 15 rats from each group were catheterized under anesthesia for measurements of vital signs. Blood samples were collected directly via heart puncture and serum was obtained by centrifugation and then refrigerated until analysis. Rats were then thoroughly perfused with iced phosphate buffered saline. Aorta and heart samples were collected after scarification. Heart samples were weighed and then cut into pieces either for fixation in formalin or storage at –80°C. The levels of SCr and BUN were measured with an autoanalyser (Hitachi Model 7070, Hitachi Electronics Co., Ltd., Tokyo, Japan).

### Immunohistochemical analysis

Aortic sections with thickness of 4 μm were used for AGE staining. Briefly, tissue sections were deparaffinized and hydrated through a series in xylene and graded alcohol (100%, 90%, 70%, and 50%). Sections were then treated with 3% H_2_O_2_/methanol and incubated in normal horse serum (3%) for 20 min at room temperature, followed by incubation with anti-AGE monoclonal antibody 6D12 (Trans Genic Inc., Kumamoto, Japan) (1:50 dilution) for 30 min at room temperature. After washing 3 times with PBS, diluted biotinylated “universal” secondary antibody (R.T.U. Vectastain Universal Elite ABC kit, Vector Laboratories Inc., Burlingame, CA) was added and incubated for another 30 min at room temperature. The density was detected using avidin-biotin-peroxidase (R.T.U. Vectastain Universal Elite ABC kit, Vector Laboratories Inc., Burlingame, CA) and diaminobenzidine (ImmPACT DAB Perxoidase Substrate, Vector Laboratories Inc., Burlingame, CA) as substrate; the sections were then counterstained with hematoxylin.

### Western blot analysis

The method used to determine collagen glycation in the aortic wall was previously described by Turk *et al*[31]. After extensive digestion with pepsin, proteinase K, and collagenase, the extracts from aortic walls were subjected to a 12% sodium dodecyl sulfate-polyacrylamide gel electrophoresis (SDS-PAGE) using a Mini PROTEANs 3 System (Bio-Rad Lab, Hercules, CA, USA). Each sample contained 40 μg/μl of protein. The gels were transferred to polyvinylidene difluoride membranes and then incubated with an anti-AGE monoclonal antibody 6D12 (1: 2500 dilution) for 60 min at room temperature, followed by using a chemiluminescence method to determine densitometry using a Dolphin-Chemi mini System (Wealtec Corp., Sparks, Nevada, USA).

### MDA measurement

The levels of MDA equivalents were determined by thiobarbituric acid reactive substances (TBARS) assay kit (Cayman Chemical Company, Ann Arbor, Michigan, USA). Briefly, samples of an aorta or left ventricle were homogenized in RIPA buffer (Sigma Chemical Co, St. Louis, MO, USA) with a 1% protease inhibitor cocktail (Sigma Chemical Co, St. Louis, MO, USA). After a brief centrifugation at 1600 × g for 10 min at 4°C, the supernatants were obtained for measuring absorbance at 540 nm.

### Statistical analyses

Results are given as means ± standard errors (SD). Two-way analysis of variance (ANOVA) was used to determine the effects of CRF and OLM on the physical properties of the rat arterial system. Simple effects analysis was used when significant interactions between CRF and OLM were found. Comparisons among means within factor levels used Tukey’s honestly significant difference method. *P* < 0.05 was considered to be significant.

## Results

In comparison with the normal control, the CRF rats yielded significant changes in renal function, arterial pressure and left ventricular hypertrophy. Apart from significantly lowering blood pressure, all the above abnormalities in CRF rats were effectively ameliorated after 8 weeks of OLM treatment (Table 1).

**Table 1:** Comparisons of body weight, renal function, blood pressure and left ventricular weight in the Wistar rat 8 weeks after subtotal nephrectomy (SNx)

	**NC (n = 13)**	**CRF (n = 14)**	**CRF + OLM (n = 14)**
	**mean ± SE**	**mean ± SE**	**mean ± SE**
BW (g)	480 ± 11.9	412.1 ± 16.6*	443.6 ± 10.5
BUN (mg/dl)	19.35 ± 0.8	64.76 ± 3.35*	46.79 ± 2.72^†^
SCr (mg/dl)	0.66 ± 0.02	1.7 ± 0.1*	1.04 ± 0.09^†^
*P*_*s*_ (mm Hg)	117.2 ± 2.2	178.5 ± 8.6*	135.7 ± 6.2^†^
*P*_*d*_ (mm Hg)	94.2 ± 2.3	126.9 ± 5.2*	101.8 ± 6.1^†^
*P*_*m*_ (mm Hg)	106.7 ± 2.3	152.1 ± 6.4*	119.2 ± 6.1^†^
*PP* (mm Hg)	23.0 ± 0.6	51.6 ± 4.4*	33.8 ± 2.2^†^
LVW (g)	0.98 ± 0.03	1.20 ± 0.04*	0.94 ± 0.05^†^
LVW/BW ( ‰)	2.04 ± 0.05	2.9 ± 0.11*	2.11 ± 0.08^†^

NC, normal controls; CRF, chronic renal failure; OLM, olmesartan.

Abbreviations: BW, body weight; P_s_, aortic systolic pressure; P_d_, aortic diastolic pressure; P_m_, mean aortic pressure; PP, pulse pressure; LVW, left ventricular weight. * : *P* < 0.05, CRF vs. NC; † :*P* < 0.05, CRF + OLM vs. CRF.

**Figure 1:**
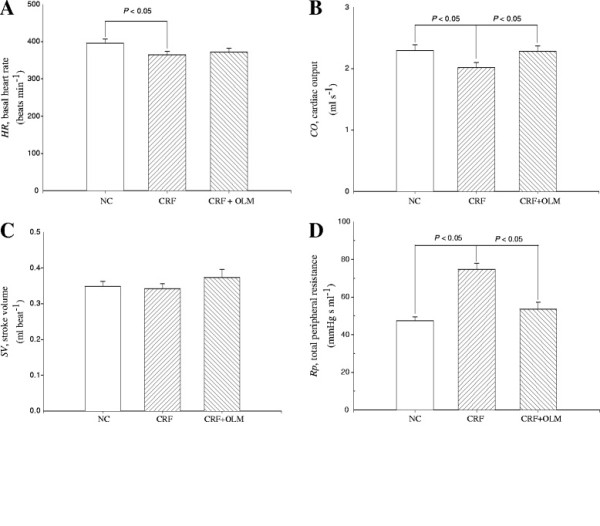
**Effects of OLM treatment on induced CRF rats and comparisons among different groups (n = 14 in each group). **HR, heart rate (**A**); CO, cardiac output (**B**); SV, stroke volume (**C**); R_p_, total peripheral resistance (**D**); NC, normal controls; CRF, chronic renal failure; OLM, olmesartan.

In comparison with normal controls, CRF rats showed significantly affected hemodynamics characterized by decreased heart rate (396.26 ± 10.76 vs. 364.30 ± 10.02 beats/min, *p* <0.05) and cardiac output (2.30 ± 0.09 vs. 2.07 ± 0.09 ml/sec, *p* <0.05) (Figure 1A, B), and conversely, a marked increase in total peripheral resistance (*p* < 0.05) (Figure 1D). The decrease in cardiac output coupled with the increase in mean aortic pressure in CRF rats (Table 1) caused a marked rise in total peripheral resistance. After 8 weeks of OLM treatment, CRF rats were normalized as evidenced by increased cardiac output (2.07 ± 0.09 vs. 2.28 ± 0.09 ml/sec, *p* <0.05) and decreased total peripheral resistance (74.56 ± 3.43 vs. 53.52 ± 3.77, mmHg sec/mL, *p* <0.05) (Figure 1B, D).

**Figure 2:**
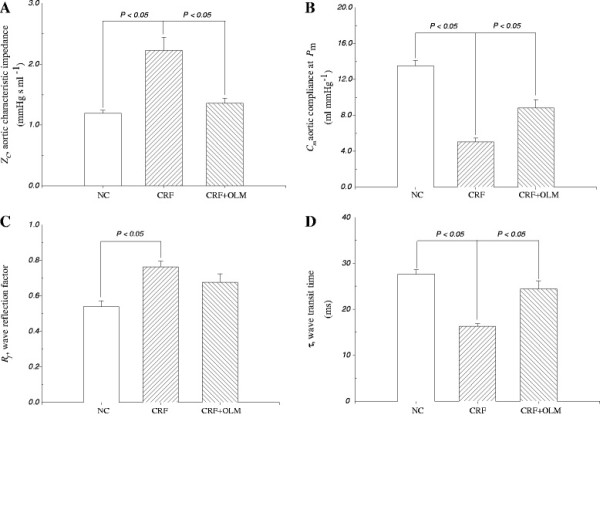
**Effects of OLM treatment on induced CRF rats and comparisons among different groups (n = 14 in each group). ***Z *_*c*_, aortic characteristic impedance (**A**); *C*_*m*_, systemic arterial compliance (**B**); *R*_*f*_ , wave reflection factor (**C**); τ, wave transit time (**D**); NC, normal controls; CRF, chronic renal failure; OLM, olmesartan.

Figure 2 depicts the aortic characteristic impedance [32] and wave reflection factor (*R*_*f*_) from the CRF rats were significantly increased than that from the controls (0.54 ± 0.03 vs. 0.76 ± 0.03 mmHg sec/ mL, *p* < 0.05) (Figure 2A, C). These changes were accompanied by the decreases of aortic compliance (*C*_*m*_) (13.50 ± 0.60 vs. 5.03 ± 0.46, *p* < 0.05, Figure 2C) and wave transit time (τ) (27.62 ± 1.02 vs. 16.26 ± 0.59 ms, *p* < 0.05, Figure 2D). Treatment with OLM showed significant effects on retarding the CRF-induced mechanical alterations in the Windkessel vessels (Table 1), as manifested by the 39% reduction in aortic characteristic impedance (*Z*_*c*_*,* 2.23 ± 0.21 vs. 1.36 ± 0.08, *p* < 0.05) and the 75.3% increase in aortic compliance (*C*_*m*_*,* 5.03 ± 0.46 vs. 8.82 ± 0.92, *p* <0.05). Early return with the augmented magnitude of the reflected wave from the peripheral circulation in CRF rats was impeded following OLM treatment, as demonstrated by the increase of 50.3% in wave transit time (τ, 16.26 ± 0.59 vs. 24.44 ± 1.76, *p* < 0.05).

There were significant changes in renal function as shown by the differences in clearances of BUN and SCr between normal rats and CRF rats (Table 1). At week 8 after the induction of CRF, the BUN was 3.3-fold and SCr 2.6-fold higher in CRF rats than the controls (*p* < 0.05), indicating an impaired renal function. We observed significant increases in the clearances of both BUN and SCr in the CRF rats following OLM administration, of which BUN decreased by 28.7% (*p* < 0.05) and SCr 38.8% (*P* < 0.05) when compared to those without treatment.

**Figure 3:**
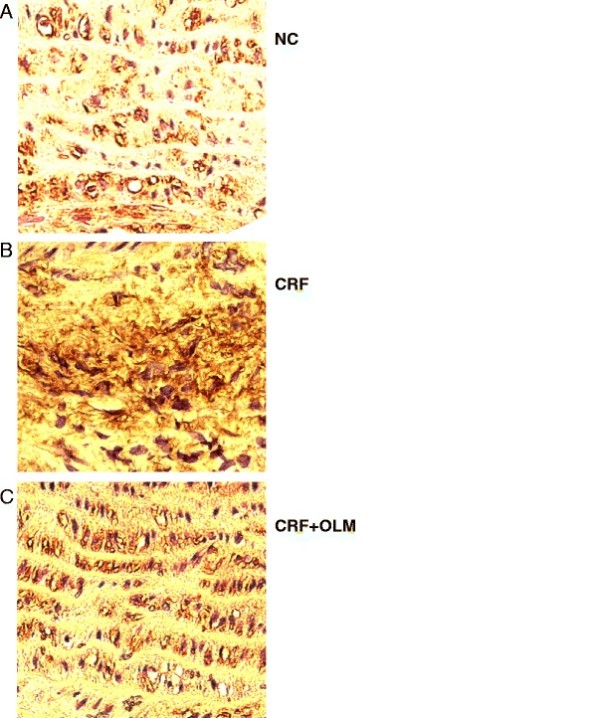
**Immunohistochemical staining for advanced glycation end products (AGEs) in the aortas at 8 weeks after SNx.** NC, normal controls; CRF, chronic renal failure; OLM, olmesartan. Magnification 400x.

**Figure 4:**
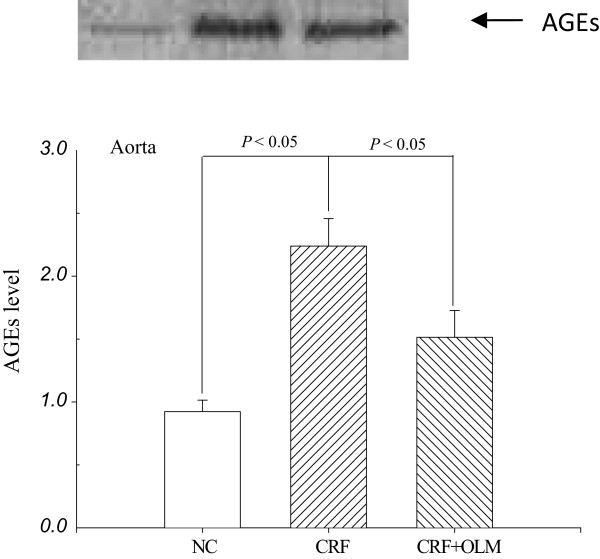
**Representative Western blot and the corresponding level of advanced glycation end products (AGEs) in the aortas of rats (n = 5) analyzed by densitometry.** Lane 1: NC; lane 2: CRF; lane 3: CRF + OLM. All data were normalized to the NC. NC, normal controls; CRF, chronic renal failure; OLM, olmesartan.

The immunointensity indicating AGE accumulation was higher in the media aortic wall of CRF rats (Figure 3), which was significantly reduced following OLM treatment for 8 weeks. Consequently, the amount of AGEs was 142% increased in collagen samples from CRF rats compared with control samples, displaying a molecular weight fragments between 26 and 34 KDa (Figure 4). After treatment with OLM for 8 weeks, AGEs decreased by 32% in glycation-derived modification of aortic collagen (*p* < 0.05).

**Figure 5:**
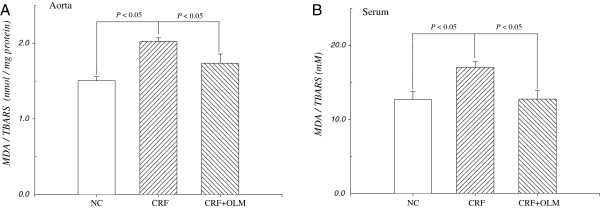
**The levels of malondialdehyde (MDA) equivalents in the aorta (A) and serum (B) measured by TBARS assay.** Increased level of MDA equivalents was observed in rats with CFR, and decreased in CRF rats (n = 12) following OLM treatment.

MDA is a biomarker of lipid-related oxidative stress, which indicates the degree of lipid peroxidation. The levels of MDA equivalents of the aorta and serum in CRF rats were markedly increased than that of controls, ranging from 1.50 ± 0.05 to 2.02 ± 0.04 nmol mg^-1^ protein (*p* < 0.05) in aorta and from 12.67 ± 1.13 to 17.01 ± 0.78 mM (*p* < 0.05) in serum (Figure 5). OLM treatment prevented CRF-induced oxidative stress in both aorta and serum, as evidenced by the reductions of levels of MDA equivalents by 14.3% and 25.1%, respectively.

## Discussion

OLM is an ARB that is characterized by potent blood pressure-lowering efficacy with a fast onset, prolonged duration of action and good tolerability. Some clinical studies have demonstrated that OLM has renoprotectve function in patients with type 2 diabetes, and beneficial effects to reduce cardiovascular risk in patients with atherosclerosis [20,33]. Recent studies have also reported that OLM is associated with a beneficial effect on lipid metabolism [34], reduction of proteinuria [35], and protection of endothelial cell from the injuries induced by oxidized LDL [36]. Our results added to the evidence that OLM exerts beneficial influences on cardiovascular disease and diabetes.

### Impacts of OLM on cardiovascular functional parameters

All the arterial pressures, including aortic systolic blood pressure, aortic diastolic pressure and mean aortic pressure, significantly increased in CRF rats (Table 1). Our data demonstrated that OLM showed significant effects on lowering these increased pressures. The pressure-lowering effect of OLM was partly due to its antagonistic actions on Angiotensin-II type 1 receptor (AT_1_R), which consequently inhibit vasoconstriction and ameliorate hypertension [37].

A decline in cardiac output and an increase in mean aortic pressure were associated with increased total peripheral resistance in CRF rats (Figure 1D). The increase in total peripheral resistance could have largely resulted from vasoconstriction and arterial stiffening. In CRF rats, ROS production via AT_1_R-mediated NADPH oxidase activation and the reaction of superoxide [38], one of the ROS, with nitric oxide (NO) generates peroxynitrite (ONOO^-^) and diminishes the vascular relaxation activity of NO [39]. Apart from activating RAS, previous studies have demonstrated that accumulated AGEs reduced NO levels, caused increased vascular smooth muscle tone [40], contributed to the cross-linking of glycated collagen in the arterial walls of CRF rats [41] and consequently led to increased arterial stiffness and total peripheral resistance. The CRF-derived physical changes in vessel resistance were ameliorated by OLM treatment, as shown by a significant reduction (28.3%) in total peripheral resistance. We therefore hypothesized that OLM treatment would improve CRF-induced vasodilatory dysfunction by blocking the action of Angiotensin II and reducing AGE formation.

Aortic characteristic impedance is frequently used as an indicator of aortic stiffness. Higher aortic characteristic impedance is associated with a stiffer aortic wall [40]. When compared to normal control rats, the aortic characteristic impedance increased (Z_c_ in Figure 2A) and wave transit time decreased (τ in Figure 2D) in CRF rats. Because the CRF-derived change in wave transit time could be a consequence of a change in pulse wave velocity [40], our results suggested that a decline in aortic distensibility had occurred in CRF rats.

The material properties of distensibility and compliance (together known as the elastic modulus) are used to describe the stiffness of a hollow vessel. Stroke volume and aortic compliance can also affect the magnitude of the pulse pressure. The arterial pulse pressure varies directly with the stroke volume, but varies inversely with the arterial compliance [40]. Our results showed increased arterial pulse pressure in CRF rats (Table 1), no significant change in stroke volume (Figure 1C) and decreased aortic compliance (Figure 2B), which suggest that the elevated arterial pulse pressure was associated with the reductions in compliance and distensibility and the aortic wall had stiffened. Decreased aortic distensibility in rats with CRF was prevented by OLM administration, as indicated by a 39% reduction in aortic characteristic impedance and a 50.3% increase in wave transit time. OLM treatment also increased the systemic arterial compliance in CRF rats by 25.3%. The improvement of the CRF-derived arterial stiffness implicated that OLM may reduce the formation of glycated collagen in the aortic wall.

Changes in timing or magnitude of the pulse wave reflection impair the loading condition of the left ventricle coupled to its arterial system [40]. Our findings of increased wave reflection factor (Figure 2C) and shortened wave transit time (Figure 2D) indicated that CRF changed the timing and magnitude of the pulse wave reflection to augment the systolic load of the left ventricle. The impaired systolic loading condition of the left ventricle caused the heart to adapt by muscular hypertrophy (Table 1). Furthermore, the increase in wave transit time indicated that OLM prevented AGE accumulation in the arterial wall collagen of CRF rats. OLM also improved the systolic loading condition of the left ventricle coupled to its vasculature system. The decreased ratio of the left ventricular weight to body weight also suggests that OLM treatment decreased vascular load and prevented CRF-related cardiac hypertrophy.

### Effects of OLM treatment on the levels of AGE and MDA

A striking increase in the AGE content of aortic collagen in CRF rats was observed in our study, suggesting that AGE-modulated collagen led to aortic stiffness. We also explored the histological evidence of considerable AGEs accumulation increased by 142% on aortic tissue of CRF rats (Figures 3C and 5C), and it was decreased by 32% after OLM treatment for 8 weeks (Figures 3D and 5). Our results that AGEs played an important role in vascular dynamics were in agreement with previous studies [42,43].

MDA is frequently measured as an indicator of lipid peroxidation and oxidative stress [44] and increased in patients with CKD [45]. Since oxidative stress was involved in the formation of AGEs [46], it might be possible to attenuate AGE formation by inhibiting several oxidative steps [11,47]. Some recent studies have proved that OLM attenuated the level of MDA equivalents and subsequently lessened the formation of AGEs in CRF rats [3,48,49]. We verified the MDA-lowering effect according to the levels of MDA equivalents decreased by 14.3% in the aorta and 25.1% in the serum after OLM treatment. Both the formation of AGEs and MDA are involved with oxidative processes producing their carbonyls or dicarbonyls. The mechanism by which OLM inhibits AGE formation was suggested to be associated with its potentials to suppress carbonyl/dicarbonyl radicals [11]. Furthermore, a previous report demonstrated that treatment of Nx rats with OLM for 8 weeks significantly reduced superoxide production [39]. Our findings were consistent with these previous studies indicating that the formation of AGEs is related to oxidative stress and might be reduced by the antioxidant ability of OLM. In addition, OLM possess renal protective properties via ameliorating progressive glomerular injury [39,50]. The findings of markedly increased excretion of BUN and SCr (Table 1) suggest that the improvement of renal function may lead to reduced AGEs accumulation in the serum of CRF rats.

## Conclusion

Our findings demonstrated that OLM provides significant protection against CRF-derived changes in the mechanical properties of blood vessels, especially the Windkessel vessels. The underlying mechanism is likely to be involved with the reduction of oxidative stress leading to subsequent decrease in the formation and accumulation of AGEs in arterial wall collagen. Other than the pressure-lowering effect of vasodilation in OLM, the inhibition of AGEs by OLM may be another pathway contributed to the improvement of the CRF-derived deterioration of blood vessels in CRF rats. The results of the current study must be also viewed in the context of many other studies of renal and cardiovascular outcomes that have shown beneficial effects of OLM.

## Abbreviations

OLM: Olmesartan; AT_1_R: Angiotensin II type 1 receptor; AGEs: Advanced glycation end products; CRF: Chronic renal failure; MDA: Malondialdehyde; CKD: Chronic kidney disease; ARBs: Angiotensin receptor blockers; ROS: Reactive oxygen species; SNx: Subtotal nephrectomy; SCr: Serum creatinine; BUN: Blood urea nitrogen; TBARS: Thiobarbituric acid reactive substances.

## Competing interests

The authors declare that they have no competing interests.

## Authors’ contributions

YCC conceived of the study, performed the experimental procedures, and drafted the manuscript. MSW and YKS provided expertise in diabetes and coordinated the research plan. KMF contributed to discussion and edited the paper. All authors contributed to results interpretation and discussion, as well as approved the final version of the manuscript.
